# The Poor Man's Home.—I

**Published:** 1897-08-21

**Authors:** 


### Modern Sociology.

### THE POOR MAN'S HOME.—I.

We need not, as some reformers do, assume that man is merely the creature of his own environment when one declares that bad surroundings tend to make bad men. Physically this is so true as to be the dullest commonplace. Morally, also, it is as well proved as moral truths can be—such truths not being susceptible to anatomical section or chemical analysis. That society at large is convinced of both is shown by the anxiety evinced by careful parents of the more favoured classes that their children should not go into the slums of a city for fear of "infection" or "contamination" —the word varies according as physical or moral taint is the more keenly feared. This anxiety is wholly just and right; the worst that one can say of it is that it does not go far enough. Even for selfish reasons the anxiety might well be keener. The fever that is bred in the slum will not remain there; it flies far and wide, and the mansion knows its ravages. The child of the slum enters the mansion in the humblest capacity, and all unconsciously breathes forth its ideas, and the children of the mansion catch, more insidiously than any fever, loose notions of honour, of decency, of purity. If the rich were wise, they would demand for the poor man's home all needful accommodation, however lacking in luxury, which would enable a family to live healthfully and decently.

And, to be honest, it must be owned that recent improvements in the homes of the working classes are due to the initiative of the richer section of the community. We are, even the most selfish of us, shocked at our brethren's poverty when it is brought home to us. The French princess who wondered that the people wanted bread when they could get cake so cheap has been subjected to ridicule for a century or more, yet doubtless, poor soul, she would have liked them to have cake and would have shared hers with them had it been possible. And since the richer classes have come to realise that the poor are uncleanly and indecent because they have no conveniences for cleanliness and decency they are willing to give them all that they need, and in many cases more than they demand.

The provision of well-built, healthy, and convenient houses for all sections of those whom we talk of in a general way as the working classes is full of difficulty. Philanthropists are apt to forget that there are so many classes within each class, and that a skilled artisan with a cleanly wife has infinitely more objection to living side by side with a casual labourer of untidy habits than a peer would have in living nextdoor to a small shopkeeper. Class dis-distinctions are as keen among the lower ten millions as among the upper ten thousand, and have often a more obvious reasonableness. Therefore, it is quite possible for a well-meant scheme for housing the poor to fail because those who promote it do not understand that all who work with their hands are not on the same social level. There are other considerations which hamper those who desire to see the working classes well housed, and which, if ignored, lead to failure. For one thing, it is sometimes stated, explicitly or impl citly, that the model dwellings for the working class erected by some wealthy individual or philanthropic society are "a charity." To imply this is almost certainly to exclude the best of the working class. It is surprising how many will cling to the shakiest garret or the dampest cellar rather than accept what they think is a charity. Yet the model dwellings will be filled. But by whom? By tenants who also regard them as a charity, who are remiss in paying their rent, and regard it as a grievance that they should have to pay rent at all, and who, especially if the rent-collector be a lady, have always a long list of woes and wrongs, meant to win sympathy of the substantial sort. In earlier experiments, moreover, houses were often ill-planned, being built in accordance with the notions of architects who did not understand the needs or the tastes of those for whom they sought to provide. Or, in country villages more often than in towns, one may see cottages, picturesque, charming to the eye, which seem at the first glance to be ideal houses. But enter them, and you will find that, having been built to the order of someone who knew more of æsthetics than of sanitation, they lack all conveniences, or are on a damp soil, or are too small to permit of a family being decently brought up in them. On the other hand, it is a mistake to make houses intended for the poorer classes too luxurious, for two reasons. One is, that they must cost so much in the building that it will not be possible, on any reasonable commercial principle, to let them at rents within the means of those for whom they are intended. And if commercial principles be left in abeyance, the other objection becomes valid. It is one which is noted by Miss Octavia Hill, who speaks from long experience. If houses with many conveniences are let too cheap, they attract the more parsimonious members of a richer class than they were meant for, who thus profit by a benevolence which was never intended for them.

But is it possible, is it right, to regard the housing of the poor as a commercial speculation? These are questions which cannot be answered by a simple "Yes," or "No." In small towns, in the country, and in the suburbs of large towns it is almost always possible to build good houses, plain, but substantial, containing rooms enough for decency, air space enough for health, and all necessary sanitary conveniences, and yet give a moderate return on the money expended. In the more central parts of large cities it is not so easy, because of the great value of land there and the cost of buying up buildings, often enough merely to knock them down. This expense was greater before the Workmen's Dwellings Act of 1890 than it is now, that Act having limited the power of landlords to demand exorbitant prices in the case of compulsory sales. At the same time the cost of land whose value has appreciated, and of buildings which are valuable by reason of their situation, will always tend to keep the speculative builder from seeking to build workmen's houses in towns. This has been the experience of the City of Glasgow. It has bought back land which it once possessed at a startling difference in price. To take only one example: In 1786 the city sold a piece of land for £58 2s. in cash and a yearly rent of £2 18s. 6d. More than a hundred years after the land w. s bought back at a cost of £11,450.

